# Intra-relation reconstruction from inter-relation: miRNA to gene expression

**DOI:** 10.1186/1752-0509-7-S3-S8

**Published:** 2013-10-16

**Authors:** Dokyoon Kim, Hyunjung Shin, Je-Gun Joung, Su-Yeon Lee, Ju Han Kim

**Affiliations:** 1 Seoul National University Biomedical Informatics (SNUBI), Div. of Biomedical Informatics, Seoul National University College of Medicine, Seoul 110799, Korea; 2 Systems Biomedical Informatics Research Center, Seoul National University, Seoul 110799, Korea; 3 Center for Systems Genomics, Pennsylvania State University, University Park, Pennsylvania, USA; 4 Department of Industrial Engineering, Ajou University, San 5, Wonchun-dong, Yeoungtong-gu, 443-749, Suwon, Korea; 5 Translational Bioinformatics Lab (TBL), Samsung Genome Institute (SGI), Samsung Medical Center, Seoul, Korea

## Abstract

**Background:**

In computational biology, a novel knowledge has been obtained mostly by identifying 'intra-relation,' the relation between entities on a specific biological level such as from gene expression or from microRNA (miRNA) and many such researches have been successful. However, intra-relations are not fully explaining complex cancer mechanisms because the inter-relation information between different levels of genomic data is missing, e.g. miRNA and its target genes. The 'inter-relation' between different levels of genomic data can be constructed from biological experimental data as well as genomic knowledge.

**Methods:**

Previously, we have proposed a graph-based framework that integrates with multi-layers of genomic data, copy number alteration, DNA methylation, gene expression, and miRNA expression, for the cancer clinical outcome prediction. However, the limitation of previous work was that we integrated with multi-layers of genomic data without considering of inter-relationship information between genomic features. In this paper, we propose a new integrative framework that combines genomic dataset from gene expression and genomic knowledge from inter-relation between miRNA and gene expression for the clinical outcome prediction as a pilot study.

**Results:**

In order to demonstrate the validity of the proposed method, the prediction of short-term/long-term survival for 82 patients in glioblastoma multiforme (GBM) was adopted as a base task. Based on our results, the accuracy of our predictive model increases because of incorporation of information fused over genomic dataset from gene expression and genomic knowledge from inter-relation between miRNA and gene expression.

**Conclusions:**

In the present study, the intra-relation of gene expression was reconstructed from inter-relation between miRNA and gene expression for prediction of short-term/long-term survival of GBM patients. Our finding suggests that the utilization of external knowledge representing miRNA-mediated regulation of gene expression is substantially useful for elucidating the cancer phenotype.

## Introduction

DNA microarrays have already been widely used for the classification of tumor subtypes or clinical outcomes for the diagnosis, treatment, or prognosis of cancer for many years [1-6]. In addition to gene expression, there have been attempts at cancer clinical outcome prediction using different levels of genomic data such as copy number, DNA methylation, or miRNA [7-11]. Despite these efforts, however, the elucidation of cancer phenotypes remains problematic since the cancer genome is neither simple nor independent but is complicated and dysregulated by multiple mechanisms in the biological system [12,13]. Previously, we have proposed a graph-based framework that integrates with multi-layers of genomic data, copy number alteration, DNA methylation, gene expression, and miRNA expression, for the prediction of clinical outcomes in glioblastoma multiforme (GBM) and serous cystadenocarcinoma [14]. The strengths of our approach were also highlighted as initiating its application using multiple scales and computation efficiency [15].

In computational biology, a novel knowledge has been obtained mostly by identifying 'intra-relation,' the relation between entities on a specific biological level such as from gene expression or from microRNA (miRNA) and many such researches have been successful [14,16,17]. However, intra-relations are not fully explaining complex cancer mechanisms because the inter-relation information between different levels of genomic data is missing, e.g. miRNA and its target genes. The 'inter-relation' between different levels of genomic data can be constructed from biological experimental data as well as genomic knowledge.

There are possible inter-relationships between the genomic features belonging to different levels of genomic data such as 'miRNA-target genes,' 'copy number alteration region-genes located in the altered region,' 'DNA methylation site-specific genes regulated by promoter regions,' etc. However, the limitation of previous work was that we integrated with multi-layers of genomic data for cancer clinical outcome prediction without considering of inter-relationship information between genomic features [14]. We assume that accuracy of prediction model increase when considering of inter-relationship between different levels of genomic data because of incorporation of information fused over genomic dataset and genomic knowledge, providing an enhanced global view on interplays in cancer mechanisms [12,18]. Therefore, when integrating multi-layers of genomic data, it will be desirable that a framework will be capable of containing the inter-relationships between genomic features belonging to different layers of the biological system.

In this paper, we propose a new integrative framework that combines genomic dataset from gene expression and genomic knowledge from inter-relation between miRNA and gene expression for the clinical outcome prediction as a pilot study. miRNAs are involved in the post-transcriptional regulation of genes either by inducing degradation of the transcript of their multiple targets or by repressing the translation of mRNA into protein [19,20]. In addition, miRNAs regulate many genes associated with different biological processes such as development, stress response, apoptosis, proliferation, and tumorigenesis [21-25]. In order to demonstrate the validity of the proposed method, the prediction of short-term/long-term survival for 82 patients in GBM was adopted as a base task. GBM is the most common and aggressive primary brain tumor in adults [26], and notorious for its tendency to recur [27]. Despite recent advances in the molecular pathology of GBM, the underling molecular mechanisms associated with clinical outcome are still poorly understood [28].

The remainder of the paper is organized as follows. Data description and methods for prediction based on intra-relation among mRNAs and prediction based on inter-relation from miRNA to mRNA are explained in the *Materials and Methods *section. In the *Results *section, experimental results and biological implications are provided to demonstrate the validity and effectiveness of our proposed approach. Finally, we discuss the meaning of our study and future works in the last section.

## Materials and methods

### Data

Normalized datasets were retrieved from the Cancer Genome Atlas (TCGA) data portal (http://tcga-data.nci.nih.gov/). A binary classification problem was set using the survival information from patient. In the classification of *short-term or long-term survival*, 'long-term' represents samples derived from patients who survived longer than 24 months [29]. The total 82 patients' records were available across the miRNA and gene expression data sets (*N *= 82), in which 54 were short-term survival while the remaining were long-term survival.

### Retrieving mRNA targets of miRNA

There is a many-to-many relationship between miRNAs and mRNAs since a single miRNA targets multiple mRNAs or a single mRNA is targeted by multiple miRNAs. In order to get target relations between miRNA and mRNA, we used miRecords which is integrated resources of miRNA that store target interactions produced by 11 established miRNA target prediction programs [30]. We created 10 variations for predicted target pairs between miRNA and genes by considering the number of positive voters from the included algorithms by miRecords (Additional file 1). Since most of the evaluation results from these variations were largely comparable, the most representative variation # 6 in Additional file 1 was used to describe the overall study results in the following sections.

### Prediction based on intra-relation among mRNAs

We used a graph-based semi-supervised learning (SSL) as a classification algorithm, which is a halfway learning scheme between supervised and unsupervised learning [31-34]. The graph-based SSL takes advantage of computational efficiency and representational ease for the biological system. The learning time of graph-based SSL is nearly linear with the number of graph edges while the accuracy remains comparable to the kernel-based methods that suffer from the relative disadvantage of a longer learning time [16,35]. In addition, the interpretation of biological phenomena can be improved because of the graph structure [36-38], which naturally fits into the graph based SSL.

**Figure 1:**
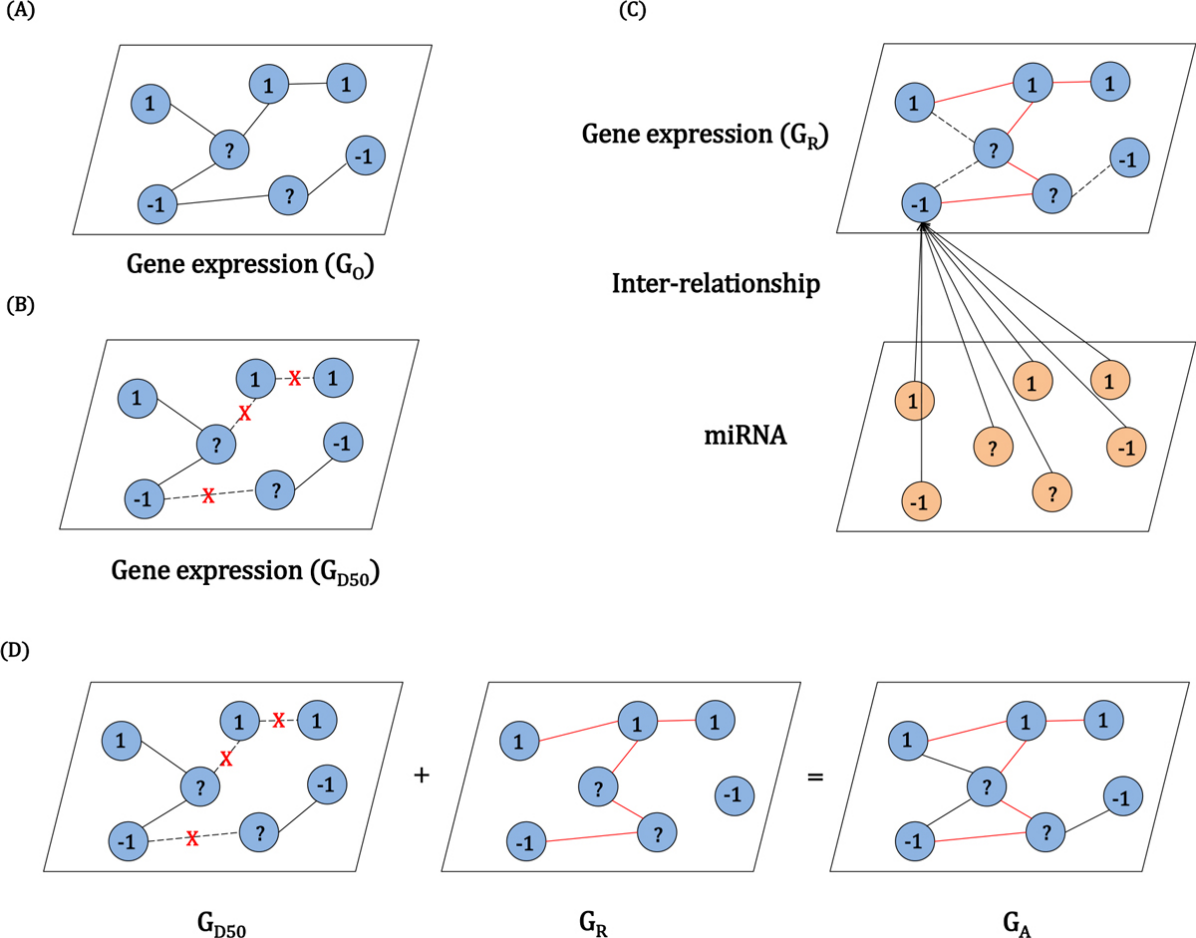
**Example model of the original, damaged, reconstructed, and augmented graphs**. (A) G_O_: Original graph from gene expression (B) G_D50_: Gene expression graph with 50 percent of damaged edges (C) G_R_: Reconstructed graph via inter-relationship between miRNA and gene expression. Red lines represent edges from inter-relation and dashed lines shows the edges from the original graph. (D) G_A_: Augmented graph by combining 50 percent of damaged graph and reconstructed graph

In this study, the entity of intra-relation or inter-relation is a patient. We define the intra-relation as a graph constructed based on single genomic data alone such as gene expression data. On the other hands, we define the inter-relation as a graph constructed based on relationship between different levels of genomic data such as gene expression and miRNA data. If two patients' samples were more closely related than to others, we assumed that the clinical outcomes of those two patients were more likely to be similar. Thus, clinical outcome prediction can be done by considering similarities between patient samples. A natural method of analyzing relationships between entities is a graph, where nodes represent patients and edges show their possible relations. Figure 1 (A) represents an example graph, which was conducted using the gene expression. An annotated patient is labeled either by '-1' or '1', indicating the two possible clinical outcomes, either 'short-term survival' or 'long-term survival.' In order to predict the label of the unannotated patient '?', the edges connected from/to the patient play an important role in influencing propagation between the patient and its neighbors. This idea can be easily formulated using graph-based semi-supervised learning [34]. Edges represent relations, more specifically similarities between patients that may be extracted from different genomic data of gene expression or miRNA. Different types of data produce different graphs. Consequently, clinical outcome prediction can benefit by integrating diverse graphs from genomic data or genomic knowledge, rather than relying only on single genomic data that may have possible limitations, i.e. incomplete information and noise. Technically, the data-setup of our experiment for the binary classification can be rephrased as ${\left\{x_{n},y_{n}\right\}}_{n=1}^{N}$ where $x_{n}\in R^{d}$ (*d *is the number of features and *N *is the number of patients) and $y_{n}\in \left\{-1,1\right\}$.

***Graph-based semi-supervised learning ***In the graph-based SSL, a patient *x_i _*(*i *= 1, ..., *n*) is represented as a node *i *in a graph, and the relationship between patients is represented by an edge. The edge strength from each node *j *to each other node *i *is encoded in element *w_ij _*of a n×n symmetric weight matrix *W*. A Gaussian function of Euclidean distance between patients was used to state connection strength:

(1) $$w_{ij}=\left\{\begin{array}{cc} \text{exp}\left(-\frac{{\left(x_{i}-x_{j}\right)}^{T}\left(x_{i}-x_{j}\right)}{\sigma^{2}}\right) & \text{if}\;\;\;i\sim j,\\ 0 & \;\text{otherwise}. \end{array}\right)$$

Nodes *i*, *j *are connected by an edge if *i *is in *j*'s *k*-nearest-neighborhood or vice versa. The labeled nodes have labels *y_l _*∈ {-1, 1}, whereas the unlabeled nodes have zeros *y_u _*= 0. An output of graph-based SSL is an *n*-dimensional real-valued vector *f *= [*f_l_*^T^*f_u_*^T^]^T ^= (*f*_1_, ..., *f_l_*, *f*_*l*+1_, ..., *f*_*n *= *l+u*_)^T^, which can be thresholded to create label predictions on *f_l _*= *f*_1_, ..., *f_n _*after learning. Graph-based SSL consists of two main conditions, which are loss condition and smoothness condition. It is assumed that *f_i _*should be close to the given label *y_i _*in labeled nodes as a loss condition, and overall, *f_i _*should not be too different from the *f_i _*of adjacent nodes as a smoothness condition. One can obtain *f *by minimizing the following quadratic functional [31,33,34]:

(2) $$\underset{f}{\text{min}}{\left(f-y\right)}^{T}\left(f-y\right)+\mu f^{T}Lf$$

where *y*=(*y*_1_, ..., *y_l_*, 0, ... 0)^T^, and the matrix *L*, called the graph Laplacian matrix [39], is defined as *L *= *D *- *W *where *D *= diag(*d_i_*), *d_i_*= ∑*_j_w_ij_*. The parameter *µ *trades off loss versus smoothness. The solution of this problem is obtained as

(3) $$f={\left(I+\mu L\right)}^{-1}y$$

where *I *is the identity matrix.

### Prediction based on inter-relationship from miRNA to mRNA

**Figure 2:**
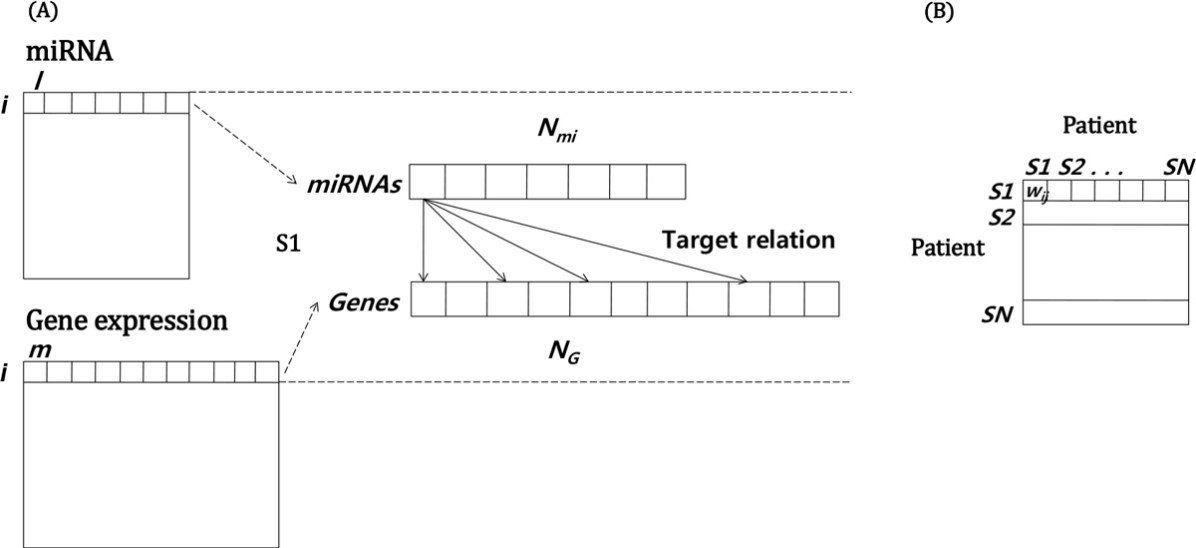
**Graphical data description**. (A) Data structure of miRNA, gene expression and their target relation (B) Similarity matrix containing inter-relation between miRNA and gene expression

The main problem of this study is to develop an adequate measure to calculate the similarity matrix containing inter-relationship information between miRNA and gene expression. There are many measures to construct the similarity matrix for graph-based semi-supervised learning such as *k*-NN graphs, ε -NN graphs, tanh-weighted graphs, exp-weighted graphs, etc [32]. For these methods, there is an assumption that the length of vector from two matrices or matrix itself should be same in order to calculate the similarity. However, it is difficult to calculate the similarity matrix containing inter-relationship information between miRNA and target genes because the length of vector from two matrices is different, for example 534 miRNAs and 12,043 genes in miRNA and gene expression, respectively (Figure 2 (A)). Thus, a new measure for calculating the similarity matrix containing inter-relationship information from different levels of genomic data has been developed in this study (Figure 2 (B)).

MicroRNA dataset is represented by *i *patients (*i *= 1, ..., *N*) and *l *miRNAs (*l *= 1, ..., *N_mi_*) and gene expression dataset is represented by *j *patients (*j *= 1, ..., *N*) and *m *genes (*m *= 1, ..., *N_G_*) (Figure 2 (A)). The edge strength from each miRNA patient to each gene expression patient is encoded in element *w_ij _*of an *N*x*N *weight matrix. A weight matrix containing inter-relationship information between miRNA and target genes is obtained by

(4) $$f_{ij}=\overset{N_{mi}}{\underset{l=1}{\sum }}\overset{N_{G}}{\underset{m=1}{\sum }}miRNA\left(i,l\right)∙gene\left(j,m\right)$$

where *m*-th gene is targeted by *l*-th miRNA. After calculating *f_ij_*, each element is normalized and transformed by

(5) $$Z_{ij}=\frac{f_{ij}-\bar{f}}{std\left(f\right)}$$

(6) $$w_{ij}=\frac{1}{1+e^{-Z_{ij}}}$$

***Integration of multiple graphs ***In order to combine the graph from gene expression and the reconstructed graph via inter-relationship, two graphs can be integrated from finding optimum combination coefficients. Information from each graph is regarded as partially independent from and partly complementary to others. Reliability might be improved by integrating all available heterogeneous data using the method proposed by Tsuda *et al. *(2005), which has been re-validated on the extended problem of protein function classification [17] and clinical outcome prediction using multi-levels of genomic data [14]. Based on the method, the integration of multiple graphs was conducted through finding an optimum coefficient of the linear combination for the individual graphs. This corresponds to finding the combination coefficients *α *for the individual Laplacians of the following mathematical formulation:

(7) $$\underset{\alpha }{\text{min}}y^{\text{T}}{\left(I+\overset{K}{\underset{k=1}{\sum }}\alpha_{k}L_{k}\right)}^{-1}y,\;\underset{k}{\sum }\alpha_{k}\leq \mu$$

where *K *is the number of graphs and *L_k _*is the corresponding graph-Laplacian of graph *G_k_*. Similar to the output prediction for single graphs, the solution is obtained by

(8) $$f={\left(I+\overset{K}{\underset{k=1}{\sum }}\alpha_{k}L_{k}\right)}^{-1}y.$$

### Experimental setting

In order to evaluate the effect of inter-relation betwee n miRNA and target genes, the intra-relation of gene expression was reconstructed from inter-relation between miRNA and gene expression. We defined the 4 cases of graph for demonstrating the validity of the proposed method (Figure 1).

(A) Original graph from gene expression (G_O_): We made an original graph from gene expression data where nodes depict patients and edges represent their possible relations.

(B) Damaged graph from the original graph (G_D_): We randomly reduced the edges from the original graph, G_O_, in order to make the incomplete graph. G_D50 _means the gene expression graph with 50 percent of damaged edges.

(C) Reconstructed graph via inter-relationship (G_R_): Reconstructed graph of gene expression was generated via inter-relationship between miRNA and gene expression.

(D) Augmented graph (G_A_): An augmented graph was generated by combining damaged graph (G_D_) from the original graph and reconstructed graph (G_R_) from inter-relation.

Since genomic data sources are generally high dimensional and noisy, and contain many redundant features, which may incur computational difficulty and low accuracy, a Student *t*-test based feature selection method was used [40]. Even though there are many feature selection techniques such as filter, wrapper, and embedded method [41], a simple univariate feature selection method was used in order to emphasize not the effect of feature selection but the effect of integration with inter-relationship between miRNAs and target mRNAs.

## Results

The receiver operating characteristic (ROC) curve plots sensitivity (true positive rate) as a function of 1-specificity (false positive rate) for a binary classifier system as its discrimination threshold is varied [42]. For each problem, we calculated area under the curve (AUC) of ROC as a performance measure. Each experiment is repeated three times in order to estimate the variance of the measurement values and five-fold cross-validation was conducted in order to overcome over-fitting. The Wilcoxon signed-rank test was used to assess the significance level of difference in performance between the results of damaged graphs and augmented graphs [43].

### Experimental results

**Figure 3:**
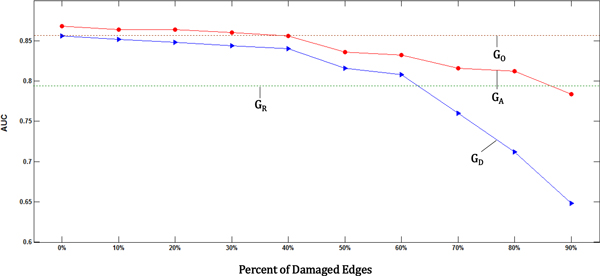
**Performance comparison of 4 cases of graphs**. G_O_: Original graph from gene expression (brown-dashed line), G_D_: Gene expression graph with damages (blue line), G_R_: Reconstructed graph via inter-relationship between miRNA and mRNA (dark green-dashed line), G_A_: Augmented graph by damaged graph and reconstructed graph (red line)

Figure 3 shows the prediction performance on the classification of short-term and long-term survival for 4 cases of proposed graphs. The AUCs of the 4 graphs (original graph from gene expression data (G_O_), damaged graph from the original one (G_D_), reconstructed graph via inter-relation between miRNA and mRNA (G_R_), and augmented graph by damaged graph and reconstructed graph (G_A_)) are shown in the *y *axis and the percent of damaged edges are represented in the *x *axis. The main result of our study is that the prediction performance was improved by integrating the original gene expression (G_O_) and the reconstructed graph via inter-relation between miRNA and mRNA (G_R_) (Figure 3). We found that the opportunity for success in prediction of clinical outcomes in GBM was increased when the prediction was based on the integration of genomic data and genomic knowledge based on inter-relationship.

As the percent of damaged edges in gene expression graph increased, the AUCs of damaged graph (G_D_) are getting decreased sharply compared to the original graph from gene expression data (G_O_) (Figure 3). However, the performances of the augmented graph (G_A_) showed robust results even though 90 percent of edges were reduced from the original graph. The performance of G_A_, a graph combining biological experimental data and genomic knowledge, is higher than the one of G_O_, an original graph from gene expression only, from 0 to 30 percent of damaged edges (Figure 3). This suggests that genomic knowledge is complementary to the prediction power of explaining cancer phenotype even though biological experimental data such as gene expression has incomplete information.

**Figure 4:**
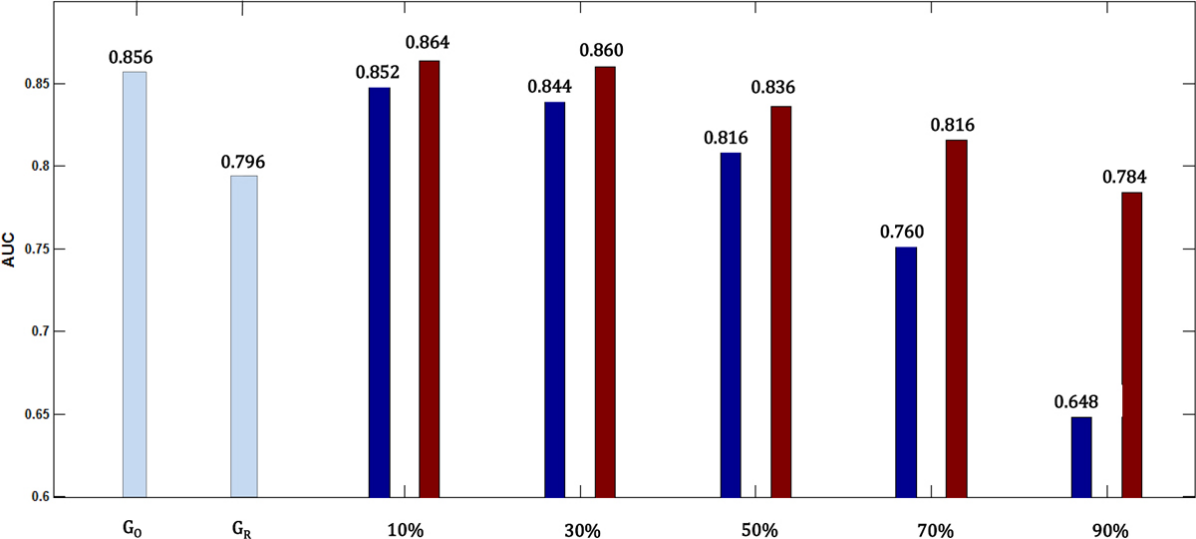
**Improving performance from the augmented knowledge based on inter-relation between mRNA and miRNA**. Dark blue bars represent the results from damaged graph and brown bar represents the one from augmented graph. Light blue bars indicate the AUCs of the original graph and reconstructed graph, respectively

The significance level of difference in performance between the results of damaged graph and augmented graph was conducted using Wilcoxon signed-rank test (Table 1). The level of significance increased as long as the percentage of damaged edges increased. Figure 4 shows a gradual increase in AUC by augmented graph. Dark blue bar represents the results from damaged graph and brown bar depicts the one from augmented graph. Light blue bars indicate the AUC of the original graph and reconstructed graph, respectively. This provides improving performance from the augmented knowledge based on inter-relation between mRNA and miRNA.

**Table 1:** Significance test of the performances between G_D _and G_A_

Percent of damaged edges	AUC of G_D_	AUC of G_A_	P-value
10%	0.852	0.864	1.80e-03
30%	0.844	0.860	2.10e-03
50%	0.816	0.836	1.91e-04
70%	0.760	0.816	2.38e-04
90%	0.648	0.784	2.36e-05

### Biological implication

**Figure 5:**
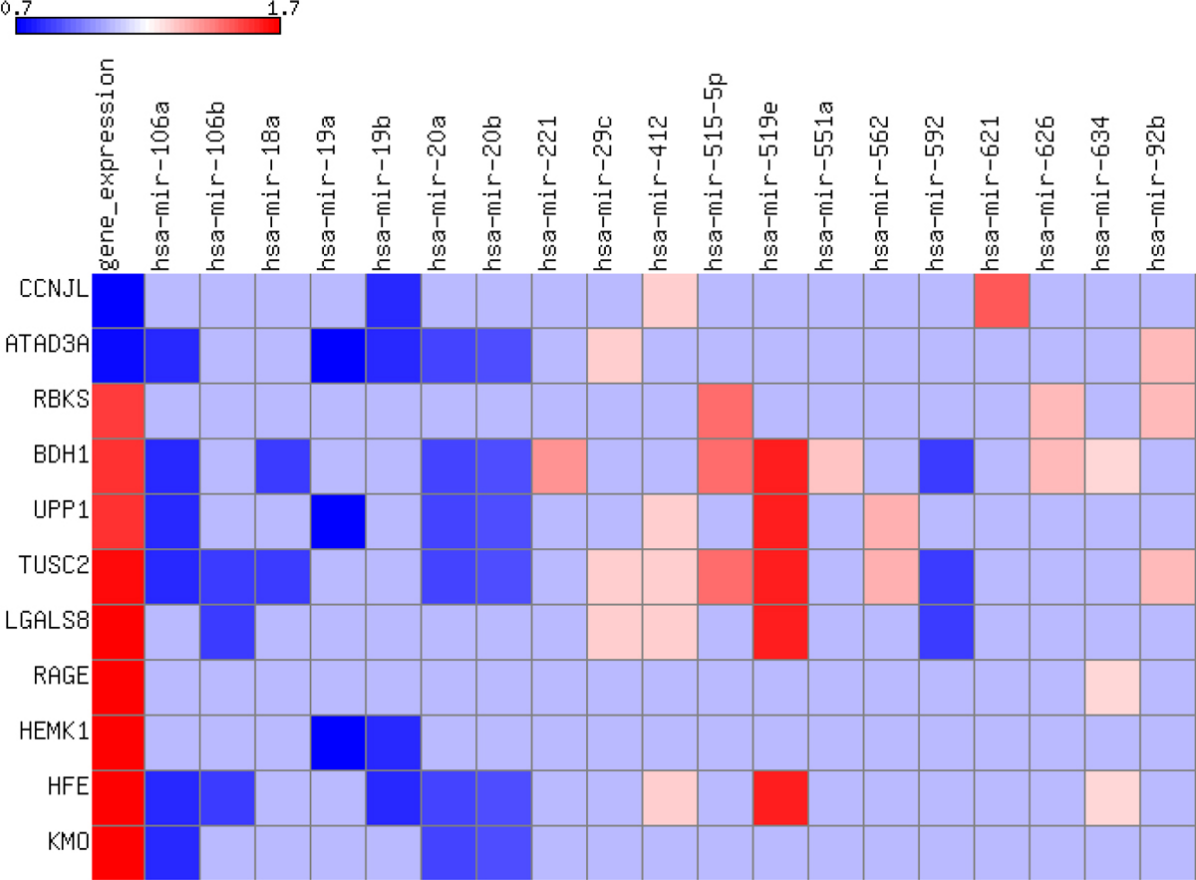
**Heatmap of selected miRNA and target gene pairs**. The first column shows the fold changes of gene expression from selected 11 genes and remaining columns represent the fold changes of miRNA expression from selected 19 miRNAs. Blue cells indicate that gene expression or miRNA expression in the short-term survival group is under-expressed compared to the long-term survival group. Light blue cells represent non-target relation between miRNA and gene.

Through the proposed model, the molecular signatures of miRNA and target genes, most associated with survival, were selected. First, miRNAs and gene features were separately selected from the prediction model based on intra-relation using independent data set, miRNA expression and gene expression, respectively. Then, miRNA and target gene pairs were selected from the prediction model based on inter-relation between miRNA and gene expression data. Figure 5 represents a heatmap of fold changes of selected miRNAs and genes, which are also belonging to selected miRNA-target gene pairs. The first column of Figure 5 shows the fold changes of gene expression from selected 11 genes and remaining columns represent the fold changes of miRNA expression from selected 19 miRNAs. Blue cell in the figure indicates that gene expression or miRNA expression in the short-term survival group is under-expressed compared to the long-term survival group. Light blue cell in the heatmap represents non-target relation between miRNA and gene. Many of these miRNA and target gene pairs affect critical biological processes that are frequently dysregulated in cancer.

For instance, three miRNAs, hsa-mir-20a, hsa-mir-106a, and hsa-mir-221, were also identified as miRNA signatures that predicts survival in Glioblastoma [44]. Hsa-mir-20a and hsa-mir-106a miRNAs were classified into the protective class and hsa-mir-221 was classified into the risk class in the previous study as well [44]. The protective miRNAs were expressed at a higher level in the long-term survival group compared to the short-term survival group while the risky miRNAs were expressed at a higher level in the short-term group than in the long-term group. The risky and protective class of these miRNAs supports the fact that their functions being either promoting or inhibitory, respectively. Under-expression of hsa-mir-106a has been shown to be associated with poor patient survival in colon cancer and glioma [45,46]. Target genes of hsa-mir-106a, *BDH1*, *UPP1*, *TUSC2*, and *KMO*, were over-expressed in the short-term survival group, which is a reverse pattern of expression in hsa-mir-106a. These genes play important roles that affect metabolic process, cell cycle, or nucleotide catabolic process in several cancers [47-50]. The miRNA cluster, which contains hsa-mir-20a, was found to promote lung cancer growth in vitro, activated by c-myc and promote tumor angiogenesis [51]. *HFE*, one of the selected target genes of hsa-mir-20a, has been found to be associated with immune response in GBM and ovarian cancer [50,52]. Among selected miRNA and target gene pairs, other pairs were of interest because they could suggest some novel indirect mechanisms in GBM tumorigenesis.

Table 2 describes the selected gene features between short-term and long-term survival group. These gene lists were sorted by the *AUC_diff*, which calculated the difference between the original AUC with 11 gene features and the AUC without one gene among 11 gene features. The high value of *AUC_diff *means that the contribution of the gene feature, being excluded for calculating the *AUC_diff*, to the prediction model is high. *RAGE *showed the highest *AUC_diff*, 0.028, and *AUC_diff *of ATAD3A, 0.024, was secondly high among gene features (Table 2).

**Table 2:** Description of the selected gene features between short-term and long-term survival group in GBM

Gene	Region	Function	Up/down	AUC_diff
RAGE	14q32.31	Renal tumor antigen/threonine kinase activity/transferase activity	Up	0.028
ATAD3A	1p36.33	ATP binding/nucleotide binding	Down	0.024
HEMK1	3p21.31	DNA binding/N-methyltransferase activity	Up	0.012
KMO	1q43	Integral to membrane/kynurenine 3-monooxygenase activity	Up	0.012
RBKS	2p23.2	D-ribose metabolic process/ribokinase activity	Up	0.012
CCNJL	5q33.3	Nucleus/regulation of progression through cell cycle	Down	0.008
LGALS8	1q43	Extracellular space/sugar binding	Up	0.008
UPP1	7p12.3	Cytoplasm/nucleoside metabolic process/nucleotide catabolic process	Up	0.008
BDH1	3q29	3-hydroxybutyrate dehydrogenase activity/metabolic process/mitochondrial inner membrane/mitochondrial matrix	Up	0.004
HFE	6p22.1	Antigen processing and presentation/ immune response/ protein complex assembly	Up	0.004
TUSC2	3p21.31	Cell cycle/cell proliferation/cell-cell signalling/negative regulation of progression through cell cycle	Up	0.000

The gene lists in the first column were sorted by the *AUC_diff*, which calculated the difference between the original AUC with 11 gene features and the AUC without one gene among 11 gene features.

The *RAGE *pathway may play an important role in *STAT3 *induction in glioma-associated microglia and macrophages, a process that might be mediated through S100B [53]. In addition, the under-expression of *ATAD3A *may be involved in the chemosensitivity of oligodendrogliomas and the transformation pathway [54].

### Comparison with other proposed methods for inter-relationship matrix

Despite the difficulty of developing an adequate measure to calculate the similarity matrix containing inter-relationship information between miRNA and gene expression, we implemented 4 measures, G_R__1, G_R__2, G_R__3, and G_R__4, and compared with the proposed method, G_R__5, in order to assess the benefit of the proposed one. G_R__1 was calculated by multiplication of correlation matrices from gene expression and miRNA expression. The method of G_R__2 was generated through the simple addition of two vectors, genes and miRNAs, for containing inter-relationship. On the other hand, the method of G_R__3 was calculated by removing miRNAs and genes, which were not belonging to the target relations, after simple addition of two vectors, genes and miRNAs. G_R__4 was focused on a targeted gene and considered multiple miRNAs targeting the specific gene when calculating the inter-relationship. In contrast to G_R__4, G_R__5, the proposed method in our study, was focused on a miRNA and considered multiple target genes from the specific miRNA.

**Figure 6:**
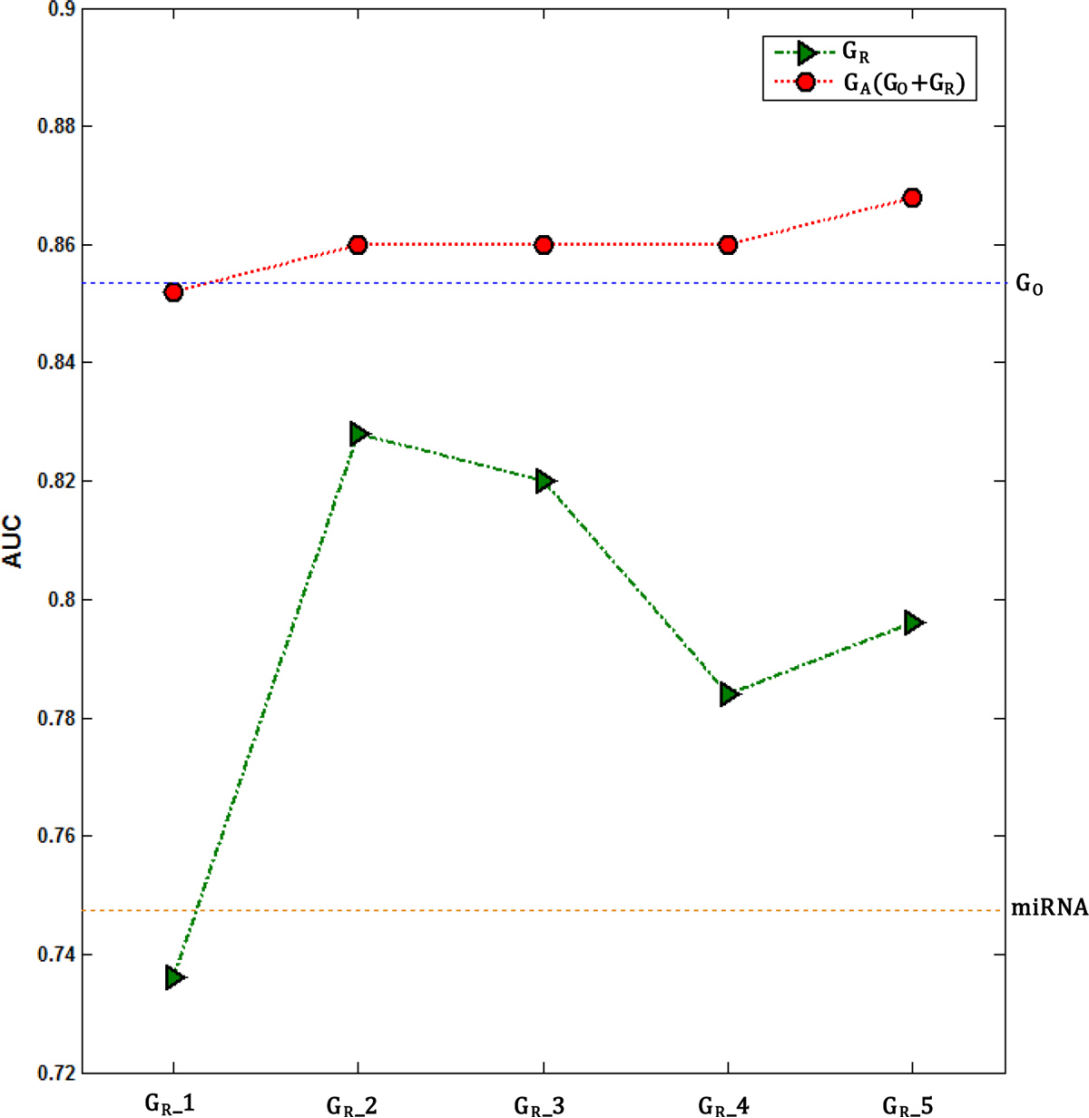
**Comparison of other proposed methods**. Four measures, G_R__1, G_R__2, G_R__3, and G_R__4, were implemented and used for calculating G_A _in order to assess the effect of the proposed method, G_R__5. The blue dotted line shows the AUC of original gene expression (G_O_) and the orange dotted line represents the AUC of miRNA data alone.

Even though the performance of G_R__2 itself showed the best (AUC = 0.828), the performance of G_A _(AUC = 0.868), integrating G_O _(AUC = 0.856) and G_R__5 (AUC = 0.796), showed the best in our comparison scheme (Figure 6). This suggests that the method of G_R__5 has more partly complementary to the gene expression itself than the others so that it improves the prediction power when integrating with gene expression.

## Conclusions

In the present study, the intra-relation of gene expression was reconstructed from inter-relation between miRNA and gene expression for prediction of short-term/long-term survival of GBM patients in order to provide a preliminary insight on the question that is how informative inter-relationship between miRNA and gene expression is when different levels of genomic dataset and valid genomic knowledge are available. Based on our results, the accuracy of our predictive model increases because of incorporation of information fused over genomic dataset from gene expression and genomic knowledge from inter-relation between miRNA and gene expression. New evidence suggests that genomic knowledge is complementary to the prediction power of explaining cancer phenotype even though biological experimental data such as gene expression has incomplete information. In addition, our finding suggests that the utilization of external knowledge representing miRNA-mediated regulation of gene expression is substantially useful for elucidating the cancer phenotype since miRNAs regulate many genes associated with different biological processes such as development, stress response, apoptosis, proliferation, and tumorigenesis.

The present study underpins our on-going work. It is expected that the next attempt will be more focused on how to utilize the information from 'intra-relation', the relation between different levels: from the genome level to epigenome, transcriptome, proteome, and further stretched to the phenome level. There might be other possible intra-relations between different layers of genomic data such as 'copy number alteration region - genes located in the alteration region,' 'DNA methylation site - specific genes regulated by promoter regions,' *etc*. Thus, when integrating multi-levels of genomic data, it might be valuable that a framework will be capable of containing the inter-relationships between genomic features belonging to different layers of the biological system as genomic knowledge. Even though this study is limited to the prediction of short-term/long-term survival in GBM as a base task, the proposed framework can be applied to other cancer types or other clinical outcomes such as grade, stage, metastasis, *etc*. In addition, we could apply the proposed method to another layer of 'intra-relation' based on miRNA expression profiles together with 'intra-relation' between mRNAs.

Recently, TCGA has been generating the additional cancer genomic data for about 20 to 25 tumor types as the second phase of the project. With abundance in different types of genomic, clinical data and valid genomic knowledge, our proposed framework will be valuable for explaining the underlying tumorigenesis, eventually leading to more effective screening strategies and therapeutic targets in many types of cancer.

## Competing interests

The authors declare that they have no competing interests.

## Authors' contributions

DK and HS designed and developed the study and wrote the manuscript. SL and JJG provided the experimental results and interpreted the results. HS and JHK provided intellectual guidance and mentorship and wrote the manuscript. All authors read and approved the final manuscript.

## Supplementary Material

Additional file 1 Supplemental table Click here for file

## Declarations

The publication cost for this work was supported by the National Research Foundation of Korea (NRF) grant funded by the Korea government (MSIP) (No. 2010-0028631).

This article has been published as part of *BMC Systems Biology *Volume 7 Supplement 3, 2013: Twelfth International Conference on Bioinformatics (InCoB2013): Systems Biology. The full contents of the supplement are available online at http://www.biomedcentral.com/bmcsystbiol/supplements/7/S3.
