# Supplementary material for: Intra-relation reconstruction from inter-relation: miRNA to gene expression
Source: BMC Syst Biol. 2013 Oct 16;7(Suppl 3):S8. doi: 10.1186/1752-0509-7-S3-S8 (PMC3852212; doi:10.1186/1752-0509-7-S3-S8)
Supplement: Additional file 1 — Supplemental table [file 1752-0509-7-S3-S8-S1.docx]

**Supplementary information**

**Supplementary Table 1.** Variation for predicted miRNA-gene target pairs

| Index | No. of algorithms showing positive voting | Numbers of | | |
| --- | --- | --- | --- | --- |
|  |  | miRNAs | Target links | Genes |
| # 1 | 3 algorithms | 553 | 1,234,390 | 17,602 |
| # 2 | 4 | 535 | 272,505 | 15,278 |
| # 3 | 5 | 407 | 53,041 | 9,747 |
| # 4 | 6 | 159 | 9,691 | 2,783 |
| # 5 | 7 | 29 | 68 | 66 |
| # 6 | 3 ~ 11 | 553 | 1,569,741 | 17,636 |
| # 7 | 4 ~ 11 | 535 | 335,351 | 15,422 |
| # 8 | 5 ~ 11 | 408 | 62,846 | 9,851 |
| # 9 | 6 ~ 11 | 159 | 9,805 | 2,816 |
| # 10 | 7 ~ 11 | 40 | 114 | 104 |
